# Evaluation of polymeric films and streptokinase for prevention of postoperative intra-abdominal adhesion in rabbits

**DOI:** 10.1590/acb415226

**Published:** 2026-09-11

**Authors:** Luisa Gouvêa Teixeira, Helena Cristina Delgado Brito, Bruna Nunes Teixeira, Julielton de Souza Barata, Rozana da Rocha Adams, Letícia Abrahão Anai, Fernanda Martinato, Andresa Matsui, Rossana Mara da Silva Moreira Thiré, Áureo Evangelista Santana

**Affiliations:** 1 Universidade Federal da Bahia – School of Veterinary Medicine and Animal Science – Salvador (BA) – Brazil.; 2 Universidade Estadual Paulista – School of Agricultural and Veterinarian Sciences – Jaboticabal (SP) – Brazil.; 3 Universidade Federal do Rio de Janeiro – Program of Metallurgical and Materials Engineering – Rio de Janeiro (RJ) – Brazil.

**Keywords:** Biocompatible Materials, Intestines, Carboxymethylcellulose Sodium, Polyvinyl Alcohol

## Abstract

**Purpose:**

To evaluate the effectiveness of polymer-based strategies for preventing intra-abdominal adhesions after small colon enterotomy and enterorrhaphy in a rabbit experimental model.

**Methods:**

Twenty-four female New Zealand White rabbits were randomly allocated into four groups (n = 6): film composed of a blend of sodium carboxymethylcellulose and polyvinyl alcohol (NaCMC-PVA); NaCMC-PVA-streptokinase (STP) film; intra-abdominal STP infusion; and no treatment. Hematological parameters were assessed preoperatively and at three and seven days postoperatively. Macroscopic evaluation of intra-abdominal adhesions and histopathological analysis of the enterorrhaphy site were performed on postoperative day 7. Results were compared within and between experimental groups.

**Results:**

Treated groups exhibited fewer hematological alterations compared with the control group. No statistically significant differences were observed among groups regarding adhesion severity or histopathological scores (*p*> 0.05). The NaCMC–PVA film showed the lowest absolute incidence of adhesions and did not impair intestinal remesothelialization.

**Conclusion:**

Based on the biological findings from this study, the NaCMC–PVA film exhibited acceptable handling characteristics and biological compatibility. These findings suggest its potential as a mechanical barrier for preventing abdominal adhesion. However, further studies with larger sample sizes and longer follow-up periods are required to confirm its effectiveness.

## Introduction

Peritoneal adhesions are defined as abnormal fibrinous bands formed within the abdominal cavity that connect organs, tissues, or both that are normally separate^1^. The development of intestinal adhesions may lead to intestinal constriction, incarceration, volvulus, or increased mesenteric tension, thereby predisposing patients to intestinal obstruction and abdominal pain^2,3^. Peritoneal adhesions occur when the parietal or visceral peritoneum is damaged, exposing the basement membrane of the mesothelial layer to surrounding tissues. Such injury, resulting from surgery, infection, or irritation, triggers a local inflammatory response^4^, leading to increased vascular permeability and the formation of a fibrin-rich inflammatory exudate composed of serum and inflammatory cells within the abdominal cavity^5^.

The reported incidence of adhesions after abdominal surgery in humans ranges from 55 to 66%, and a proportion of these patients require additional surgical treatment^6^. Rabbits are also highly susceptible to adhesion formation^7^ and are therefore widely used as experimental models for adhesion studies^8^.

Adhesion prevention can be achieved by modifying the pathophysiological mechanisms involved in its formation, in conjunction with meticulous surgical technique. The main objectives of abdominal adhesion prevention include minimizing serosal inflammation, maintaining or enhancing peritoneal fibrinolytic activity, mechanically separating surfaces with adhesiogenic potential, and stimulating intestinal motility^3,5^.

To this end, several therapeutic strategies have been investigated, including pharmacological approaches such as the use of anti-inflammatory agents^9^, drugs that interfere with the coagulation cascade, such as heparin^10^, and fibrinolytic agents, including streptokinase (STP). STP is produced by β-hemolytic *Streptococcus* and plays an important role as a plasminogen activator^4,11,12^. More recent studies have focused on physical barrier methods that use devices of various morphologies composed of natural or synthetic polymers to physically separate peritoneal surfaces^13-17^. Some approaches combine both strategies through the use of drug delivery devices. Despite extensive research efforts and the availability of several commercial products, an ideal anti-adhesion treatment has not been developed yet.

Polyvinyl alcohol (PVA) is a synthetic polymer widely used in medical applications because of its biocompatibility, mechanical strength, and film-forming properties^14,18^. Sodium carboxymethylcellulose (NaCMC) is a natural anionic, water-soluble polymer commonly employed in biomedical applications due to its availability, high viscosity, low cost, non-toxicity, and biocompatibility^19^. Experimental use of these polymers has reduced abdominal adhesion formation following peritoneal instillation of PVA gel in rabbits^20^, NaCMC gel in ponies^21^, and PVA-NaCMC gel in pigs^22^. However, complete prevention of adhesion formation was not achieved. To date, no studies have reported the use of polymeric films composed of NaCMC-PVA or NaCMC-PVA-STP for the prevention of abdominal adhesions.

The aim of this study was to evaluate the effects of STP infusion and the use of polymeric films containing NaCMC-PVA or NaCMC-PVA-STP on abdominal adhesion formation, as well as on hematological and anatomopathological parameters, following enterotomy and enterorrhaphy of the descending colon in a rabbit experimental model.

## Methods

### Production of NaCMC-PVA and NaCMC-PVA-STP films

NaCMC (molecular weight ~250,000) and PVA (molecular weight = 85,000–124,000; > 99% hydrolyzed) were obtained from Sigma Aldrich (United States of America). The films were produced by solvent casting technique, according to the methodology described by Oliveira et al.^23^, with minor modifications. Briefly, two polymeric solutions were prepared: a 16 wt% PVA solution, dissolved in distilled water at 90°C under constant agitation for four hours, and a 4 wt% NaCMC solution, dissolved in distilled water at room temperature under magnetic stirring at 800 rpm for eight hours. The two solutions were subsequently mixed at a 1:1 ratio using magnetic stirring at 600 rpm for 20 minutes. To produce the NaCMC-PVA-STP films, 99,000 IU of STP (streptase 1,500,000 IU; CSL Behring, Brazil) was added to the polymeric blend. The final solutions were poured into polystyrene molds and allowed to dry by solvent evaporation in a ventilated oven at 25°C for 12 hours. After drying, films measuring 2 × 3 cm were exposed to ultraviolet (UV) radiation for 1 hour and individually packaged for subsequent use.

### Animals

All animal procedures were approved by the Ethics Committee on the Use of Animals of the Universidade Estadual Paulista “Júlio de Mesquita Filho” (UNESP), under protocol no. 11,684/16, and were conducted in accordance with established guidelines for animal research.

Twenty-four female New Zealand White rabbits (*Oryctolagus cuniculus*), aged between 120 and 180 days old and weighing 3.46 ± 0.49 kg (mean ± standard deviation), obtained from the vivarium of UNESP (SP, Brazil), were used in this study. Throughout the experimental period, the animals were housed in the cuniculture sector and received daily commercial pelleted feed for rabbits (Agromix, Brazil), ramie (*Boehmeria nivea*), coast-cross hay (*Cynodon dactylon*), and water *ad libitum*. Prior to surgery, all rabbits underwent a 30-day quarantine and acclimatization period to local environmental conditions, handling, and feeding.

### Anesthesical and surgical procedures

Rabbits were randomly allocated into four groups of six animals each, and all experimental groups were operated on in parallel. All animals underwent the same anesthetic, surgical, and postoperative protocols, including celiotomy, enterotomy, and enterorrhaphy of the descending colon. The groups differed only regarding the intraoperative methods used to prevent abdominal adhesion formation:

- Group 1 (G1): a NaCMC-PVA film was applied;
- Group 2 (G2): a NaCMC-PVA-STP film was used;
- Group 3 (G3): 99,000-IU STP was infused into the abdominal cavity;
- Group 4 (G4): negative control, receiving no polymeric film implantation or STP infusion.

**Figure 1:**
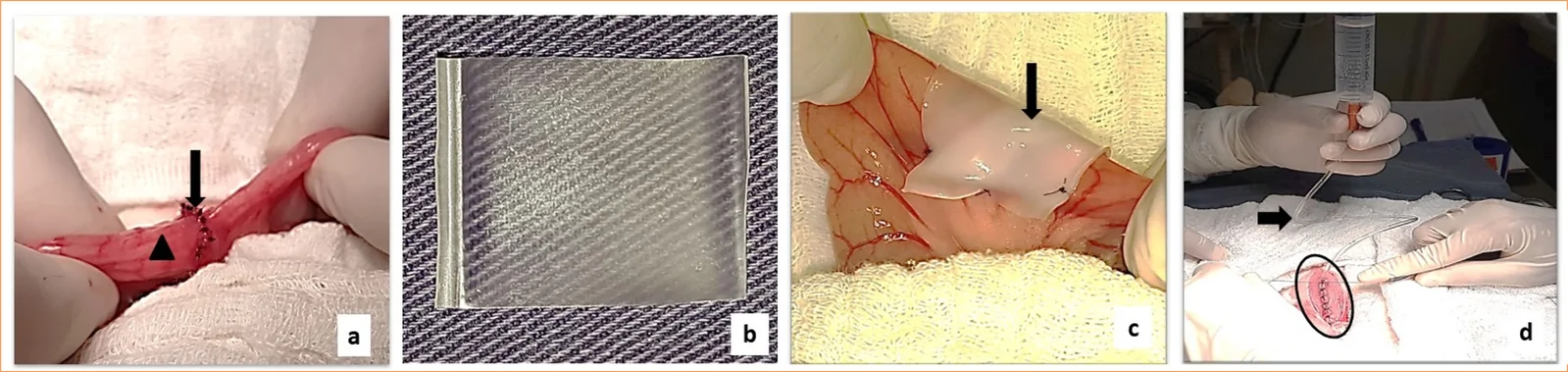
Celiotomy of rabbits. (a) Transverse enterorrhaphy using a simple interrupted suture pattern (arrow) on the antimesenteric border of the descending colon (arrowhead) in the control group (G4). (b) NaCMC-PVA film showing transparency before swelling. (c) Swollen NaCMC-PVA film (arrow) covering the enterorrhaphy site in group 1. (d) Intra-abdominal streptokinase infusion in group 3 using a urethral catheter (arrow) attached to a syringe after enterorrhaphy and closure of the abdominal midline (ellipse).

NaCMC: sodium carboxymethylcellulose; PVA: polyvinyl alcohol film. Source: Elaborated by the authors.

As preanesthetic medication, acepromazine (0.05 mg/kg) and morphine (1 mg/kg) were administered intramuscularly. Anesthetic induction was performed with intravenous propofol (6 mg/kg). Anesthesia was maintained with 2% isoflurane delivered by inhalation. A ventral midline abdominal incision was performed, the descending colon was exteriorized, and a 0.5-cm longitudinal enterotomy was created on the antimesenteric border. Enterorrhaphy was performed transversely using a simple interrupted suture pattern with polyglecaprone 25 USP 5-0 (Fig. 1a). After completion of the enterorrhaphy, the treatment protocols differed according to the experimental group.

In G1, the NaCMC-PVA film (Fig. 1b) was immersed in sterile 0.9% sodium chloride solution for 45 seconds and fixed to the seromuscular layer of the descending colon using six simple interrupted sutures of polyglecaprone 25 USP 5-0, fully covering the enterorrhaphy site (Fig. 1c). The same procedure was performed in G2 using the NaCMC-PVA-STP film. In G3, animals received an intra-abdominal infusion of 99,000 IU of STP diluted in 20 mL of sterile 0.9% sodium chloride solution immediately after enterorrhaphy (Fig. 1d). Animals in G4 did not receive any adhesion prevention treatment. The abdominal wall was closed in layers: the musculature using a Sultan suture pattern with nylon USP 2-0, the subcutaneous tissue using a zig-zag pattern with polyglecaprone 25 USP 5-0, and the skin using a simple continuous pattern with nylon USP 2-0. All animals were monitored for seven days after surgery.

Postoperatively, flunixin meglumine (1 mg/kg, intramuscularly), metoclopramide hydrochloride (1 mg/kg, orally), and glucose (500 mg/kg, orally) were administered twice daily for three consecutive days. Ringer’s lactate solution (70 mL/kg, subcutaneously) was administered twice daily for five days, and enrofloxacin (5 mg/kg, subcutaneously) was administered twice daily for seven days.

At the end of the experimental period, all rabbits were euthanized using intramuscular acepromazine (0.05 mg/kg), followed by intravenous propofol (10 mg/kg) and an overdose of sodium pentobarbital (100 mg/kg, intravenously). Death was confirmed by the absence of corneal reflexes and cardiorespiratory arrest.

### Blood tests

Blood samples were collected by jugular venipuncture immediately before celiotomy (T0), and at three (T1) and seven days (T2) after surgery. Complete blood count and total plasma protein determination were performed using blood samples collected into tubes containing ethylenediaminetetraacetic acid (EDTA). Red blood cell count, total leukocyte count, hemoglobin concentration, packed cell volume, and platelet count were determined by an automated hematology analyzer (ABC Vet Animal Blood Counter; Horiba Instruments, United States of America). Differential leukocyte counts were performed on blood smears stained with May–Grünwald–Giemsa and methanol. Total plasma protein concentration was measured by manual refractometry (Master SUR/N Alpha; Atago, Brazil). Blood samples collected into sodium citrate tubes were analyzed using a coagulometer (Clot Quick Timer II; Drake, Brazil) to determine prothrombin time (PT; Hemostasis 501-5/4; Labtest Diagnóstica, Brazil), activated partial thromboplastin time (aPTT; Hemostasis 502-1/4; Labtest Diagnóstica, Brazil), and fibrinogen concentration (Fibrinogen 506-4/2; Labtest Diagnóstica, Brazil).

### Macroscopic and histopathological study

On the seventh postoperative day, the abdominal cavity of all rabbits was macroscopically evaluated for the presence and severity of intra-abdominal adhesions, as well as for other macroscopic healing-related changes. Adhesion severity scores (ASS) were graded on a scale from 0 to 4^24^ (Table 1). The absorption of the polymeric films was specifically evaluated in G1 and G2.

**Table 1:** Classification of adhesions severity observed during macroscopic evaluation of the abdominal cavity of rabbits.

Adhesion severity score	Description
0	No adhesion
1	Unique and easily separable adhesion
2	Adhesions in small extension and that break with small traction
3	Extensive visceral adhesions that extend to the abdominal wall
4	Numerous, extensive, and dense adhesions involving the mesentery, intestine, omentum, and abdominal wall

Source: adapted of Knightly et al.^24^.

Samples of the descending colon encompassing the enterorrhaphy site were collected *en bloc* from all experimental groups and fixed in 10% buffered formalin for subsequent histopathological analysis. Histopathological scoring was performed to quantify inflammatory infiltrate, neovascularization, necrosis, edema, and fibrin deposition in sections stained with hematoxylin and eosin. Collagen deposition was evaluated in sections stained with Masson’s trichrome. The scoring system was defined as follows:

- 0 = absent;
- 1 = slight increase;
- 2 = moderate increase;
- 3 = marked increase.

All macroscopic and microscopic evaluations were performed by investigators blinded to the surgical procedures.

### Statistical analysis

Parametric data obtained from the hematological evaluations were analyzed using analysis of variance, followed by Tukey’s post hoc test to compare means between different time points within each group and among groups. Parametric data are presented as mean ± standard deviation. Non-parametric data were analyzed using the Kruskal-Wallis’ test to compare histopathological scores and ASS among the experimental groups. To compare the prevalence of adhesions among groups, a generalized linear model was applied. The response variable was modeled using a Bernoulli distribution with a logit link function, and analysis of deviance was performed under a completely randomized design. The presence of adhesions was coded as 1, and absence as 0. Statistical significance was set at *p* < 0.05. All analyses were performed using R.

## Results

The produced films were transparent in the dry state (Fig. 1b). Immersion in sterile 0.9% sodium chloride solution immediately before use as a barrier rendered the films opaque and more flexible, facilitating handling and suturing by the surgeon. Comparatively, NaCMC-PVA-STP films were less flexible than NaCMC-PVA films after immersion in saline. The mean surgical time was 97 minutes for G1 (NaCMC-PVA film), 86 minutes for G2 (NaCMC-PVA-STP film), 78 minutes for G3 (intra-abdominal STP administration), and 68 minutes for G4 (no treatment). Only rabbits in G3 exhibited mild bleeding through the skin sutures approximately 20 minutes after intraperitoneal STP infusion, which ceased spontaneously.

Evaluation of the hematological profile of G1 (Table 2) and G2 (Table 3) showed that, when T0 was compared with subsequent time points, there was a reduction (*p* < 0.05) in red blood cell count, hemoglobin concentration, and packed cell volume at T1 and T2. In G2, basophil counts increased (*p* < 0.05) at T1 and T2, monocyte counts increased (*p* < 0.05) at T2 only, and prothrombin time decreased (*p* < 0.05) at T2. In G3 (Table 4), red blood cell counts and packed cell volume decreased (*p* < 0.05) only at T1, while prothrombin time decreased (*p* < 0.05) at both T1 and T2. In G4 (Table 5), a reduction (*p* < 0.05) in red blood cell count was observed at T1 and T2, packed cell volume decreased (*p* < 0.05) at T2, eosinophil counts increased (*p* < 0.05) at T1, and monocyte counts increased (*p* < 0.05) at T2. In this group, PT decreased (*p* < 0.05) at T1 and T2, whereas aPTT increased (*p* < 0.05) at T1.

**Table 2:** Values obtained for blood count, total plasma protein, fibrinogen, prothrombin, and activated partial thromboplastin times in the blood of rabbits from group 1 (film of sodium carboxymethylcellulose and polyvinyl alcohol)<tfn href="tfn01">*</tfn>.

Parameters	T0	T1	T2
RBC (x10^6^/μL)	6.35 ± 0.62^a^	4.87 ± 0.46^b^	4.84 ± 0.58^b^
Hemoglobin (g/dL)	13.85 ± 0.87^a^	10.96 ± 0.98^b^	11.01 ± 1.35^b^
PCV (%)	40.55 ± 4.05^a^	31.20 ± 2.80^b^	31.70 ± 3.64^b^
Total leukocythes (/μL)	3,450.00 ± 662.57^a^	5,900.00 ± 1,902.63^a^	4,683.33 ± 2,413.64^a^
Basophils (/μL)	58.33 ± 61.27^a^	125.00 ± 95.23^a^	197.33 ± 189.52^a^
Eosinophils (/μL)	16.50 ± 29.25^a^	23.50 ± 38.86^a^	34.83 ± 35.01^a^
Band neutrophils (/μL)	0 ± 0^a^	0 ± 0a	0 ± 0^a^
Heterophils (/μL)	1,323.50 ± 330.00^a^	2,524.83 ± 1,424.62^a^	1,789.00 ± 1,518.98^a^
Lymphocytes (/μL)	2,045.66 ± 494.44^a^	3,220.16 ± 600.13^a^	2,634.16 ± 800.97^a^
Monocytes (/μL)	6.00 ± 14.69^a^	6.50 ± 15.92^a^	28.00 ± 43.83^a^
Platelets (x10^3^/μL)	259.16 ± 67.87^a^	295.50 ± 64.40^a^	447.66 ± 57.08^a^
Total plasm protein (g/dL)	6.13 ± 0.32^a^	5.63 ± 0.36^a^	5.66 ± 0.66^a^
PT (seconds)	7.33 ± 0.64^a^	6.90 ± 1.13^a^	7.36 ± 1.55^a^
aPTT (seconds)	15.10 ± 3.04^a^	15.98 ± 1.88^a^	15.73 ± 2.63^a^
Fibrinogen (g/L)	2.66 ± 0.33^a^	7.11 ± 2.02^b^	3.83 ± 1.01^a^

T0: immediately before sedation for celiotomy; T1: three days after celiotomy; T2: seven days after celiotomy; RBC: red blood cells; PCV: packed cell volume; PT: prothrombine time; aPTT: actived partial thromboplastine time;

* means followed by the same letters on the lines do not differ by Tukey’s test at 5% significance (*p* < 0.05). Source: Elaborated by the authors.

**Table 3:** Values obtained for blood count, total plasma protein, fibrinogen, prothrombin, and activated partial thromboplastin times in the blood of rabbits from group 2 (film of sodium carboxymethylcellulose, polyvinyl alcohol and streptokinase)<tfn href="tfn01">*</tfn>.

Parameters	T0	T1	T2
RBC (x10^6^/μL)	6.40 ± 0.60^a^	5.01 ± 0.50^b^	5.07 ± 0.32^b^
Hemoglobin (g/dL)	14.15 ± 0.98^a^	11.31 ± 0.95^b^	11.60 ± 1.10^b^
PCV (%)	40.85 ± 4.18^a^	32.65 ± 2.36^b^	33.73 ± 3.07^b^
Total leukocythes (/μL)	4,016.66 ± 818.33^a^	5,000.00 ± 761.57^a^	5,683.33 ± 617.79^a^
Basophils (/μL)	48.50 ± 96.00^b^	103.66 ± 41.46^a^	138.33 ± 82.53^a^
Eosinophils (/μL)	5.16 ± 12.65^a^	50.00 ± 56.21^a^	51.66 ± 126.55^a^
Band neutrophils (/μL)	0 ± 0^a^	0 ± 0^a^	0 ± 0^a^
Heterophils (/μL)	1,414.66 ± 289.46^a^	1,751.83 ± 609.67^a^	2,163.50 ± 596.37^a^
Lymphocytes (/μL)	2,548.33 ± 640.17^a^	3019.50 ± 794.68a	3,196.16 ± 300.36^a^
Monocytes (/μL)	0 ± 0^b^	75.00 ± 119.61^ab^	152.33 ± 190.71^a^
Platelets (x10^3^/μL)	263.66 ± 56.78^a^	273.50 ± 85.02^a^	427.16 ± 95.08^a^
Total plasm protein (g/dL)	6.53 ± 0.41^a^	6.26 ± 0.54^a^	6.06 ± 0.46^a^
PT (seconds)	7.53 ± 0.64^a^	6.73 ± 0.60^ab^	6.36 ± 0.19^b^
aPTT (seconds)	18.78 ± 4.89^a^	18.46 ± 3.05^a^	14.60 ± 3.92^a^
Fibrinogen (g/L)	2.65 ± 0.24^a^	8.48 ± 1.43^a^	6.45 ± 4.28^a^

T0: immediately before sedation for celiotomy; T1: three days after celiotomy; T2: seven days after celiotomy; RBC: red blood cells; PCV: packed cell volume; PT: prothrombine time; aPTT: actived partial thromboplastine time;

* means followed by the same letters on the lines do not differ by Tukey’s test at 5% significance (*p* < 0.05). Source: Elaborated by the authors.

**Table 4:** Values obtained for blood count, total plasma protein, fibrinogen, prothrombin, and activated partial thromboplastin times in the blood of rabbits from group 3 (streptokinase infusion into the abdominal cavity)<tfn href="tfn01">*</tfn>.

Parameters	T0	T1	T2
RBC (x10^6^/μL)	5.97 ± 0.61^a^	5.19 ± 0.59^b^	5.72 ± 0.73^a^
Hemoglobin (g/dL)	11.38 ± 3.90^a^	11.38 ± 0.97^a^	11.95 ± 0.81^a^
PCV (%)	37.51 ± 2.67^a^	33.45 ± 3.32^b^	36.40 ± 4.37^ab^
Total leukocythes (/μL)	3,683.33 ± 847.15^a^	5,416.66 ± 803.53^a^	4,783.33 ± 1,448.33^a^
Basophils (/μL)	62.16 ± 55.28^a^	46.00 ± 52.45^a^	102.83 ± 92.60^a^
Eosinophils (/μL)	19.50 ± 47.76^a^	73.00 ± 45.42^a^	25.16 ± 39.54^a^
Band neutrophils (/μL)	0 ± 0^a^	0 ± 0^a^	0 ± 0^a^
Heterophils (/μL)	1,363.16 ± 582.01^a^	1,928.83 ± 1,116.61^a^	1,628.33 ± 971.30^a^
Lymphocytes (/μL)	2,230.50 ± 362.58^a^	3,368.83 ± 582.65^a^	3,027.00 ± 1,148.39^a^
Monocytes (/μL)	66.50 ± 55.34^a^	0 ± 0^a^	0 ± 0^a^
Platelets (x10^3^/μL)	232.00 ± 25.70^a^	290.50 ± 73.72^a^	439.66 ± 62.77^a^
Total plasm protein (g/dL)	5.93 ± 0.57^a^	5.73 ± 0.20^a^	6.00 ± 0.17^a^
PT (seconds)	9.21 ± 1.38^a^	7.98 ± 1.01^b^	6.88 ± 0.24^c^
aPTT (seconds)	15.68 ± 4.14^a^	16.80 ± 2.74^a^	15.43 ± 2.53^a^
Fibrinogen (g/L)	2.40 ± 0.25^a^	5.70 ± 1.39^a^	3.58 ± 0.83^a^

T0: immediately before sedation for celiotomy; T1: three days after celiotomy; T2: seven days after celiotomy; RBC: red blood cells; PCV: packed cell volume; PT: prothrombine time; aPTT: actived partial thromboplastine time;

* means followed by the same letters on the lines do not differ by Tukey’s test at 5% significance (*p* < 0.05). Source: Elaborated by the authors.

**Table 5:** Values obtained for blood count, total plasma protein, fibrinogen, prothrombin, and activated partial thromboplastin times in the blood of rabbits from group 4 (control)<tfn href="tfn01">*</tfn>.

Parameters	T0	T1	T2
RBC (x10^6^/μL)	5.54 ± 0.47^a^	4.95 ± 0.44^b^	4.61 ± 0.39^b^
Hemoglobin (g/dL)	12.10 ± 0.97^a^	11.11 ± 0.98^a^	10.50 ± 0.88^a^
PCV (%)	35.55 ± 2.74^a^	32.10 ± 2.30^ab^	30.33 ± 2.64^b^
Total leukocythes (/μL)	4,450.00 ± 847.93^a^	7,833.33 ± 2,292.30^a^	6,000.00 ± 1,807.76^a^
Basophils (/μL)	49.00 ± 82.58^a^	34.00 ± 83.28^a^	5.33 ± 13.06^a^
Eosinophils (/μL)	25.50 ± 27.96^b^	202.83 ± 161.59^a^	18.66 ± 28.91^b^
Band neutrophils (/μL)	0 ± 0^a^	31.66 ± 77.56^a^	0 ± 0^a^
Heterophils (/μL)	2,027.00 ± 212.79^a^	4,029.50 ± 1,065.30^a^	3,305.66 ± 867.80^a^
Lymphocytes (/μL)	2,253.16 ± 832.17^a^	3,354.33 ± 1,444.77^a^	2,432.83 ± 1,477.65^a^
Monocytes (/μL)	95.33 ± 55.85^b^	181.00 ± 151.93^ab^	237.50 ± 81.27^a^
Platelets (x10^3^/μL)	272.83 ± 74.06^a^	384.33 ± 52.38^a^	396.50 ± 42.02^a^
Total plasm protein (g/dL)	5.86 ± 0.37^a^	6.10 ± 0.20^a^	5.90 ± 0.30^a^
PT (seconds)	8.46 ± 1.27^a^	7.41 ± 0.93^b^	7.26 ± 1.02^b^
aPTT (seconds)	23.66 ± 8.34^b^	29.98 ± 9.72^a^	19.31 ± 4.93^b^
Fibrinogen (g/L)	3.01 ± 0.33^a^	5.26 ± 1.60^a^	3.33 ± 0.85^a^

T0: immediately before sedation for celiotomy; T1: three days after celiotomy; T2: seven days after celiotomy; RBC: red blood cells; PCV: packed cell volume; PT: prothrombine time; aPTT: actived partial thromboplastine time;

* means followed by the same letters on the lines do not differ by Tukey’s test at 5% significance (*p* < 0.05). Source: Elaborated by the authors.

When comparing hematological parameters among the four groups at T1, basophil counts were higher (*p* < 0.05) in G1 and G2 than in G4. Monocyte counts were higher (*p* < 0.05) in G4 compared with G1 and G3. G4 also exhibited the highest values (p < 0.05) for eosinophil count and aPTT compared with the other groups. At T2, significant differences (*p* < 0.05) in basophil and monocyte counts remained similar to those observed at T1. Additionally, G2 differed (*p* < 0.05) from all other groups by presenting the shortest PT. G1 and G4 showed lower (*p* < 0.05) red blood cell counts than G3, and G4 presented the lowest packed cell volume (*p* < 0.05), differing from G3.

In animals from G1 and G2, the polymeric films were swollen, whitish, firmly adhered to the intestinal serosa at the implantation site, and showed no displacement or complete degradation seven days postoperatively. Macroscopically, NaCMC-PVA-STP films exhibited greater porosity than NaCMC-PVA films. One rabbit in G1 (16.66%) and one in G2 (16.66%) presented stenosis at the enterorrhaphy site. A small yellowish punctate area on the serosal surface of the enterorrhaphy, classified as granulomatous inflammation, was observed in one rabbit from G2 (16.66%), two from G3 (33.33%), and two from G4 (33.33%). Microscopically, these areas consisted of epithelioid macrophages and eosinophils.

The three treated groups showed a lower prevalence of intra-abdominal adhesions compared with the control group, but the differences among groups were not statistically significant (*p* = 0.33). Adhesions were observed in one rabbit in G1 (16.66%), three in G2 (50%), three in G3 (50%), and four in G4 (66.66%). Although G1 exhibited the lowest ASS, no significant differences were detected among the four groups (*p* = 0.37). Macroscopic healing changes of the abdominal cavity and ASS values are summarized in Table 6.

**Table 6:** Values related to the amount of macroscopic healing changes observed in the abdominal cavity of the rabbits at seven days after enterotomy and enterorrhaphy of the descending colon, including adhesions, stenosis, granulomas, and dehiscence.

Macroscopic healing changes	G1	G2	G3	G4
Adhesion of some organ to enterorrhaphy	0	1 (ASS = 4)	3 (ASS = 1; 2; 3)	2 (ASS = 1; 2)
Adhesion between descending colon mesentery and the bladder	0	1 (ASS = 2)	0	0
Adhesion between descending colon mesentery	1 (ASS = 2)	2 (ASS = 2; 2)	0	2 (ASS = 2; 2)
Adhesion of the uterus to the abdominal wall	0	0	0	1 (ASS = 3)
Descending colon stenosis	1	1	0	0
Granuloma in enterorrhaphy	0	1	2	2
Dehiscence of enterorrhaphy	0	0	0	0
Total healing changes	2	6	5	7

G1: group of the film containing sodium carboxymethylcellulose and polyvinyl alcohol; G2: group of the film containing sodium carboxymethylcellulose, polyvinyl alcohol, and streptokinase; G3: streptokinase infusion group in the abdominal cavity; G4: control group (no film or streptokinase infusion); ASS: adhesion severity score (0 to 4). Source: Elaborated by the authors.

**Figure 2:**
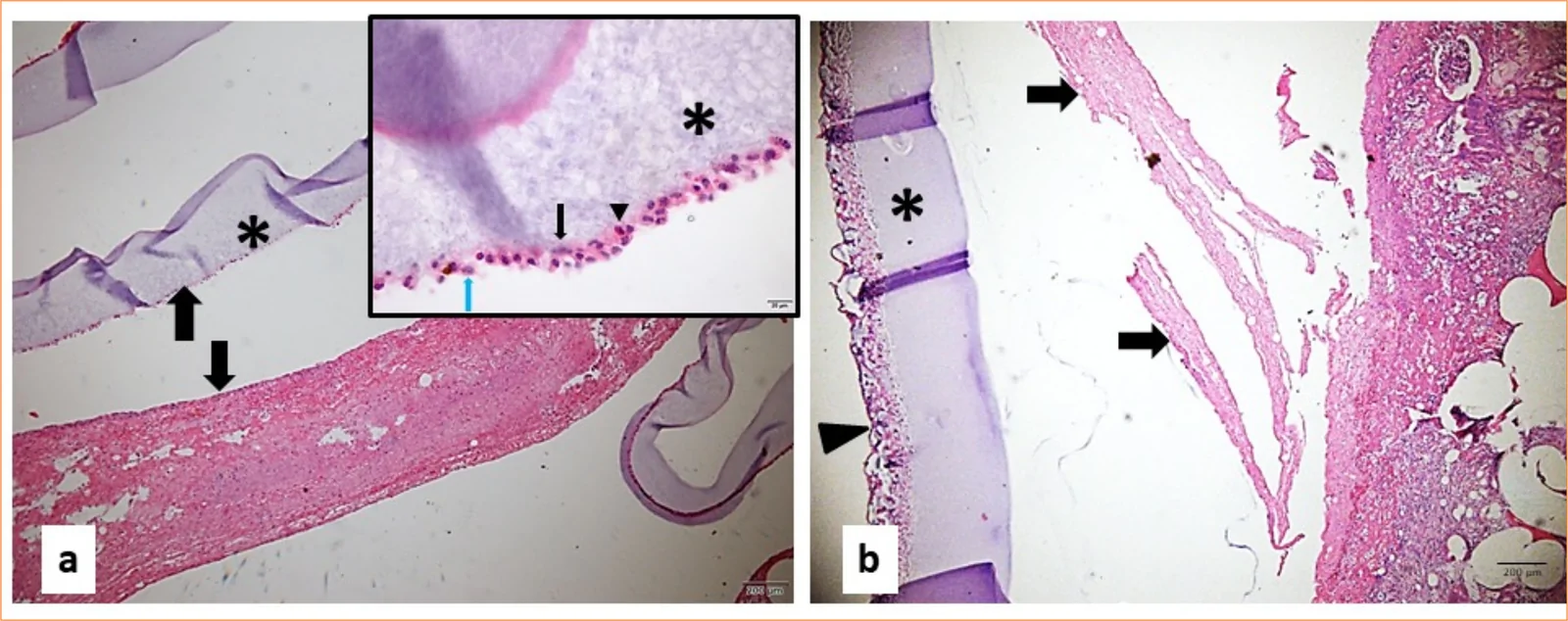
Histological images of the descending colon from rabbits treated with polymeric films. (a) At the edge (upward arrow) of the NaCMC-PVA film (*) in contact with the serosal layer of the descending colon (downward arrow), mesothelial cells are observed interspersed with inflammatory cells. Hematoxylin and eosin stain, x4; scale bar = 200 µm. (Detail) Presence of mesothelial cells (black arrow), macrophages (blue arrow), and eosinophils (arrowhead) at the edge of the film (*). Hematoxylin and eosin stain, x40; scale bar = 20 µm. (b) Presence of fibrin (arrows) between the NaCMC-PVA-STP film (*) and the serosal layer of the descending colon, with evidence of degradation on the external surface of the film (arrowhead) facing the parietal peritoneum. Hematoxylin and eosin stain, x4; scale bar = 200 µm.

NaCMC: sodium carboxymethylcellulose; PVA: polyvinyl alcohol film; STP: streptokinase. Source: Elaborated by the authors.

Microscopic analysis of descending colon samples from all groups revealed an inflammatory infiltrate predominantly composed of eosinophils and macrophages surrounding the suture material. In both biomaterial-treated groups, a hyaline substance, morphologically similar to fibrin, was observed between the intestinal serosa and the polymeric film. Only animals in G1 exhibited longitudinally aligned mesothelial cells on the surface of the film in contact with the intestinal serosa (Fig. 2a). In contrast, only animals in G2 showed irregularities on the external surface of the film, consistent with degradation (Fig. 2b).

**Figure 3:**
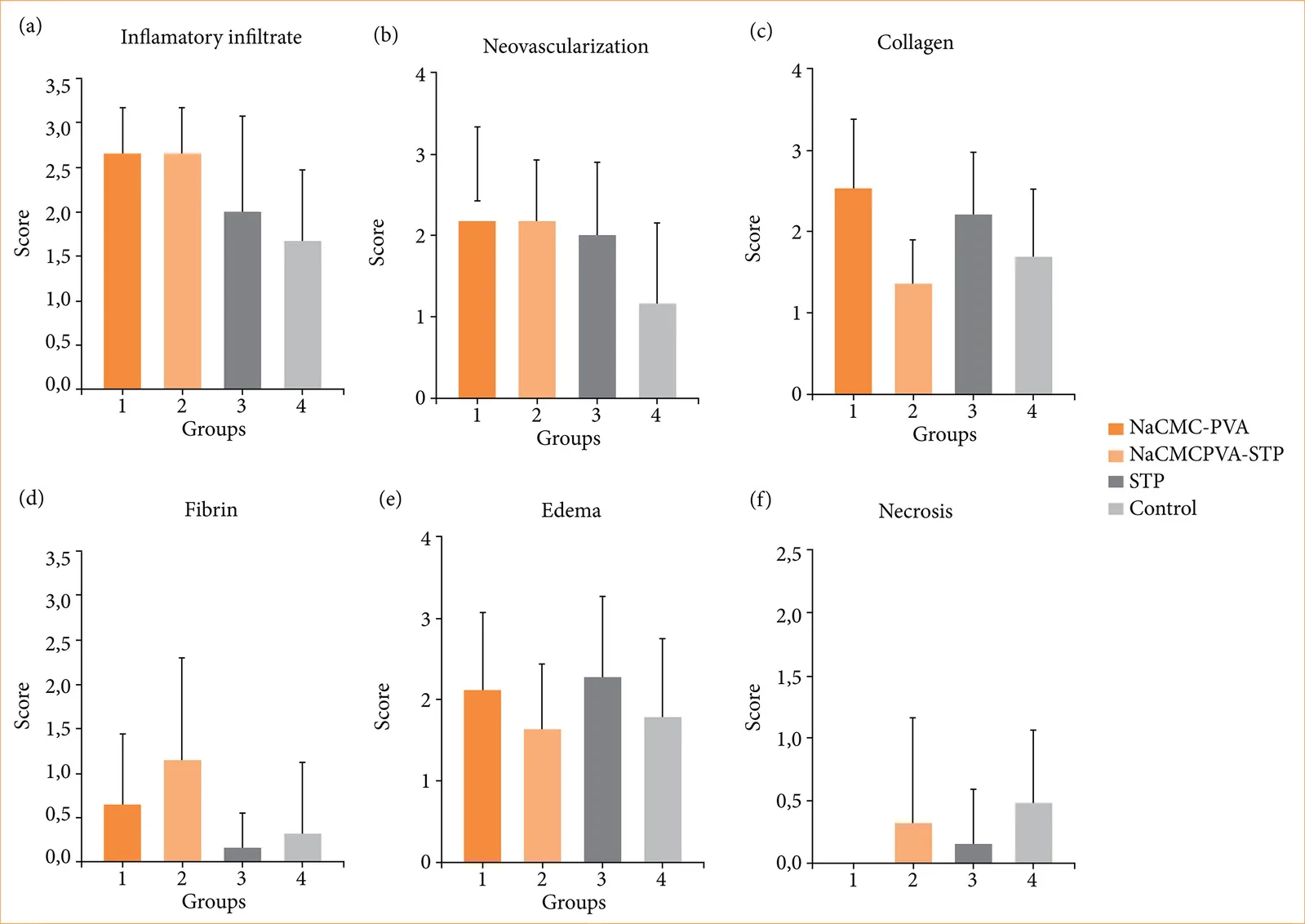
Graphical representation of semiquantitative histopathological scores (0 to 3) of the descending colon enterorrhaphy among the experimental groups. (a) Inflammatory infiltrate. Hematoxylin and eosin stain. (b) Neovascularization. Hematoxylin and eosin stain. (c) Collagen deposition. Masson’s trichrome stain. (d) Fibrin deposition. Hematoxylin and eosin stain. (e) Edema. Hematoxylin and eosin stain. (f) Necrosis. Hematoxylin and eosin stain. Scores 0: absent; 1: slight increase; 2: moderate increase; and 3: marked increase. The Kruskal-Wallis’ test was used.

NaCMC: sodium carboxymethylcellulose; PVA: polyvinyl alcohol film; STP: streptokinase; *statistically significant difference (*p* < 0.05). Source: Elaborated by the authors.

No statistically significant differences (*p* > 0.05) were detected among the four groups regarding microscopic evaluation scores. Nevertheless, numerical differences were observed: G1 and G2 exhibited the highest mean scores for inflammatory infiltrate intensity (Fig. 3a) and neovascularization (Fig. 3b); G1 presented the highest collagen deposition score (Fig. 3c); G2 showed the highest fibrin score on the external surface of the film (Fig. 3d); G3 had the highest edema score (Fig. 3e); and G4 exhibited the highest necrosis score (Fig. 3f).

## Discussion

To the best of our knowledge, this study is the first to evaluate the use of NaCMC-PVA and NaCMC-PVA-STP polymeric films for preventing intra-abdominal adhesions following enterotomy and enterorrhaphy. Previous experimental studies have investigated different materials to prevent intra-abdominal adhesions^15,16^, but none have described the application of the polymeric films evaluated in the present study.

Although commercially available mechanical barriers do not completely prevent abdominal adhesion formation, they have been shown to reduce both incidence and severity in humans^25,26^ and animals^27,28^. Different products have been developed with varying degrees of success along the translational tissue engineering research spectrum, but their clinical translation remains limited^29^. Therefore, the development of systems capable of preventing adhesion formation remains essential to improve surgical outcomes, and to reduce postoperative pain, reoperation rates, and associated healthcare costs^29,30^.

Among the evaluated treatments, the NaCMC-PVA film used in G1 showed a favorable overall profile relative to the objectives of this study. Animals in this group exhibited the lowest absolute prevalence of postoperative abdominal adhesions (*p* > 0.05), fewer hematological alterations, and no detrimental effects on intestinal healing based on microscopic evaluation, suggesting favorable biological behavior under the experimental conditions. These findings are consistent with the fundamental principle of using drug-free mechanical barriers for adhesion prevention, which are intended for intraperitoneal application to physically separate injured serosal surfaces, reducing adhesion formation while supporting normal healing and minimizing the risk of systemic or local adverse effects^30-32^.

An additional noteworthy finding was the presence of mesothelial cells aligned on the surface of the biomaterial in contact with the intestinal serosa in G1. This observation suggests that the NaCMC-PVA film may have acted as a scaffold for cell adhesion and proliferation, thereby contributing to colonic remesothelialization. Preservation or stimulation of remesothelialization is considered a key characteristic of an ideal mechanical barrier for adhesion prevention^33^. In this context, this behavior may reflect functional characteristics described for advanced anti-adhesion membranes, including the capacity to allow nutrient exchange and support tissue repair processes^34^.

After immersion in saline solution, the films demonstrated excellent handling properties and were easily applied and sutured to the intestinal serosa. When aqueous media penetrate the NaCMC-PVA film, swelling occurs as the polymeric network expands^23^. This swelling reduces film stiffness, increasing flexibility, while maintaining sufficient mechanical strength to allow manipulation without tearing or cracking during the surgical procedure. The mechanical integrity of the film is supported by polymer entanglements, crosslinking, and crystalline regions within the polymer matrix. While NaCMC contributes to increased plasticity and flexibility^20^, PVA enhances the mechanical strength of the final material^35^.

Due to their hydrophilic nature and high swelling capacity in aqueous environments^36,37^, NaCMC and PVA form a slippery mucilage and reduce mucoadhesive properties^38^, which were favorable characteristics for their selection in the formulation of the films. Both polymers are classified as biocompatible and biodegradable^18,19^, although the films remained present at the implantation site seven days postoperatively. Biomaterials used for postoperative anti-adhesion should present an appropriate degradation rate, since excessively slow degradation may induce inflammation and foreign body reactions, while rapid degradation may compromise their intended anti-adhesion effect^39^. Therefore, long-term studies are required to assess the complete degradation and resorption of the films developed in the present study.

Several doses and combinations of STP have been reported in the literature to prolong its *in-vivo* half-life^40^, treat thrombosis in rabbits^41^, perform cytotoxicity testing^42^, and prevent abdominal adhesions in rats^12,43^. In the present study, the mean dose of STP selected was 33,000 IU/kg^41^, applied both in the NaCMC-PVA-STP film and for intraperitoneal infusion. However, rabbits treated with STP (G2 and G3) did not demonstrate superior adhesion prevention compared with the other groups, in contrast to previous findings in rats undergoing laparotomy and peritoneal STP administration^12^.

The dose of STP administered intraperitoneally in G3 could not be increased due to the risk of severe abdominal hemorrhage. Hemorrhage from the peritoneal surface and systemic complications have been reported following intravenous STP administration at doses ranging from 33,300 to 50,000 IU/kg in rabbits^44^. Lower doses reduce the likelihood of adverse effects, and this can be achieved by incorporating the drug into a delivery system such as a polymeric film^43^. The use of the same STP dose in G2 and G3 was intended to standardize the experimental design and allow reliable comparison between treatment modalities.

The absence of peritoneal hemorrhage in animals from G2 suggests that the polymeric matrix functioned effectively as a drug delivery system, providing controlled local release of STP. Drug delivery systems are designed to minimize adverse effects associated with single high-dose administration, repeated intraperitoneal injections, or catheter-based infusion, which may predispose patients to infection^43^. In this regard, the objectives of controlled drug delivery and reduction of systemic side effects were successfully achieved in the present study.

The pharmacokinetics of STP are characterized by biphasic elimination, with an initial half-life of 11 to 17 minutes followed by a terminal phase of approximately 80 minutes, resulting in complete clearance from the peritoneal cavity within six hours^45^. Consequently, intraperitoneal administration of a single dose, as performed in G3, likely provides limited fibrinolytic activity and insufficient duration to effectively prevent adhesion formation. Sustained fibrinolytic activity for at least five days is considered necessary for optimal adhesion prevention^46^.

Regarding the hematological findings, the observed decreases (*p* < 0.05) in red blood cell count, hemoglobin concentration, and packed cell volume across all groups were attributed to intraoperative blood loss and repeated blood sampling. The increase (*p* < 0.05) in circulating monocytes observed in G2 and G4 may reflect a systemic inflammatory response^47^. These groups also exhibited more pronounced alterations in abdominal tissue repair. Monocytes are actively recruited to sites of inflammation and differentiate into various macrophage subtypes depending on the tissue environment and inflammatory stimuli^48^.

The increase (*p* < 0.05) in eosinophil counts observed exclusively in G4 may be associated with tissue remodeling following enterotomy. Eosinophils also play a role in inflammatory responses mediated by basophils and mast cells and may contribute to host tissue damage^49^. Consistent with this observation, G4 exhibited the highest histopathological scores for intestinal necrosis, although without statistical significance (*p* > 0.05), suggesting a possible relationship between these findings.

The decrease (*p* < 0.05) in prothrombin time observed in animals from G2, G3, and G4 may reflect postoperative changes in coagulation status, although mean values remained within the established reference range for female rabbits^50^. These groups also exhibited higher macroscopic adhesion formation. Although a possible association between reduced PT and adhesion development can be considered, a direct causal relationship cannot be established based on the present data.

Colonic stenosis was an adverse outcome observed in some animals treated with polymeric films in G1 and G2. This complication may be attributed to the mechanical pressure exerted by the swollen film on the descending colon, which is anatomically thin in rabbits^51^. The absence of stenosis in animals that did not receive polymeric films (G3 and G4) supports this hypothesis. Reducing film thickness may mitigate this complication in future applications.

The granulomatous inflammation observed at the enterorrhaphy site in some animals was attributed to the suture material acting as a foreign body. Such granulomas represent histiocytic reactions to inert materials, including sutures and talc from surgical gloves, and can occur during the normal healing process following enterorrhaphy^48^.

Although no statistically significant differences were observed in adhesion prevention, the film composed only of NaCMC and PVA presented favorable safety and handling characteristics and did not appear to adversely affect the hematological profile or the quantity and quality of scar tissue at the enterorrhaphy site. The ability of this film to promote mesothelial cell growth represents a potentially beneficial characteristic, suggesting a role in remesothelialization and its possible application as a mechanical barrier for preventing abdominal adhesions. However, these results should be interpreted with caution, given the limited sample size and short follow-up period, which may have reduced the statistical power of the study and did not allow for the evaluation of long-term outcomes, including complete reabsorption of the material.

Furthermore, this platform may be further explored and optimized through the incorporation of additional substances designed to enhance adhesion prevention or promote tissue healing. The manufacturing process and application strategy of this polymeric film could also be adapted for use in humans or other animal species by modifying the thickness or dimensions of the film to meet the specific anatomical and functional requirements of different organs or surgical sites.

## Conclusion

Based on the biological findings observed in this study, the film composed exclusively of NaCMC and PVA showed the lowest adhesion incidence and did not impair intestinal healing. These findings suggest acceptable biocompatibility and handling characteristics. However, the results should be considered preliminary, as further studies with larger sample sizes and longer evaluation periods are required to confirm its effectiveness.

## Data Availability

The data will be available upon request.
